# Japanese gastric cancer treatment guidelines 2014 (ver. 4)

**DOI:** 10.1007/s10120-016-0622-4

**Published:** 2016-06-24

**Authors:** Japanese Gastric Cancer Association

**Affiliations:** Japanese Gastric Cancer Association, 465 Kajii cho, Kawaramachihirokoji, Kamigyo ku, Kyoto, 602-8566 Japan

## Preface to the English edition

This English edition was made based on the Japanese version published as a book in 2014. Our policy in compiling this edition was to attempt not to include new evidence that emerged since the publication of the Japanese version so as to maintain consistency of the two editions. However, for some particularly important issues, we provided additional comments and new references reflecting the new evidence.

## Preface

Version 4 of the Japanese Gastric Cancer Treatment Guidelines was completed in May 2014, incorporating new evidence that includes those delivered as a quick bulletin in the website of the Japan Gastric Cancer Association after publication of the previous version. It remains largely conformed to the textbook style, but a new section consisting of clinical questions and answers (Q&A) was added to address some important clinical issues for which hard evidence is unavailable.

To compile this version, the guideline committee nominated several working groups, each assigned to make relevant contributions to unsolved issues on the following topics: (1) surgery and lymphadenectomy for junctional cancer, (2) clinical pathway, (3) follow-up after curative surgery, (4) treatment of technically resectable metastatic cancer, (5) risk calculation for surgical intervention and (6) treatment of cancer of the gastric remnant. Of these, tentative consensuses were reached on the first three topics that were included as new sections in the text, whereas further discussion was deemed necessary for the last two topics. The clinical importance of the fourth topic and lack of hard evidence related to that topic prompted the committee to establish a Q and A section to provide tentative best answers to important clinical questions on technically resectable metastatic cancer.

Major points of revision in the current version are listed below:

1. The section on types and definitions of gastric surgery has been revised.
2. An algorithm showing the tentative standard of the extent of lymphadenectomy that can be recommended for junctional cancer less than 4 cm in diameter has been presented.
3. Laparoscopic distal gastrectomy for clinical stage I cancer was upgraded from an investigational treatment to an option in general practice.
4. Chemotherapeutic regimens were classified into three recommendation categories based on the level of evidence and consensus among the committee members.
5. A revision was made to the definition of curative resection among tumors of expanded indication for endoscopic resection. Additional descriptions were given on the biopsy-derived scar and component of “muc” in the submucosa of the endoscopy-resected specimen.
6. Clinical questions were raised on treatment strategy for technically resectable metastatic cancer and chemotherapy for patients for whom evidence-based standard treatment may not be applicable, and the tentative but best possible answers were provided.
7. Exemplary samples of the clinical pathway for management of patients who underwent gastrectomy and the follow-up schedule after surgery for gastric cancer were presented.

The description of tumor status (T/N/M and stage) in this guideline remains to be based on the third English edition of the Japanese Classification of Gastric Carcinoma [1], which is identical to that in the 7th edition of the International Union Against Cancer (UICC)/TNM.

## Treatments

### Algorithm of standard treatments to be recommended in clinical practice

**Fig. 1:**
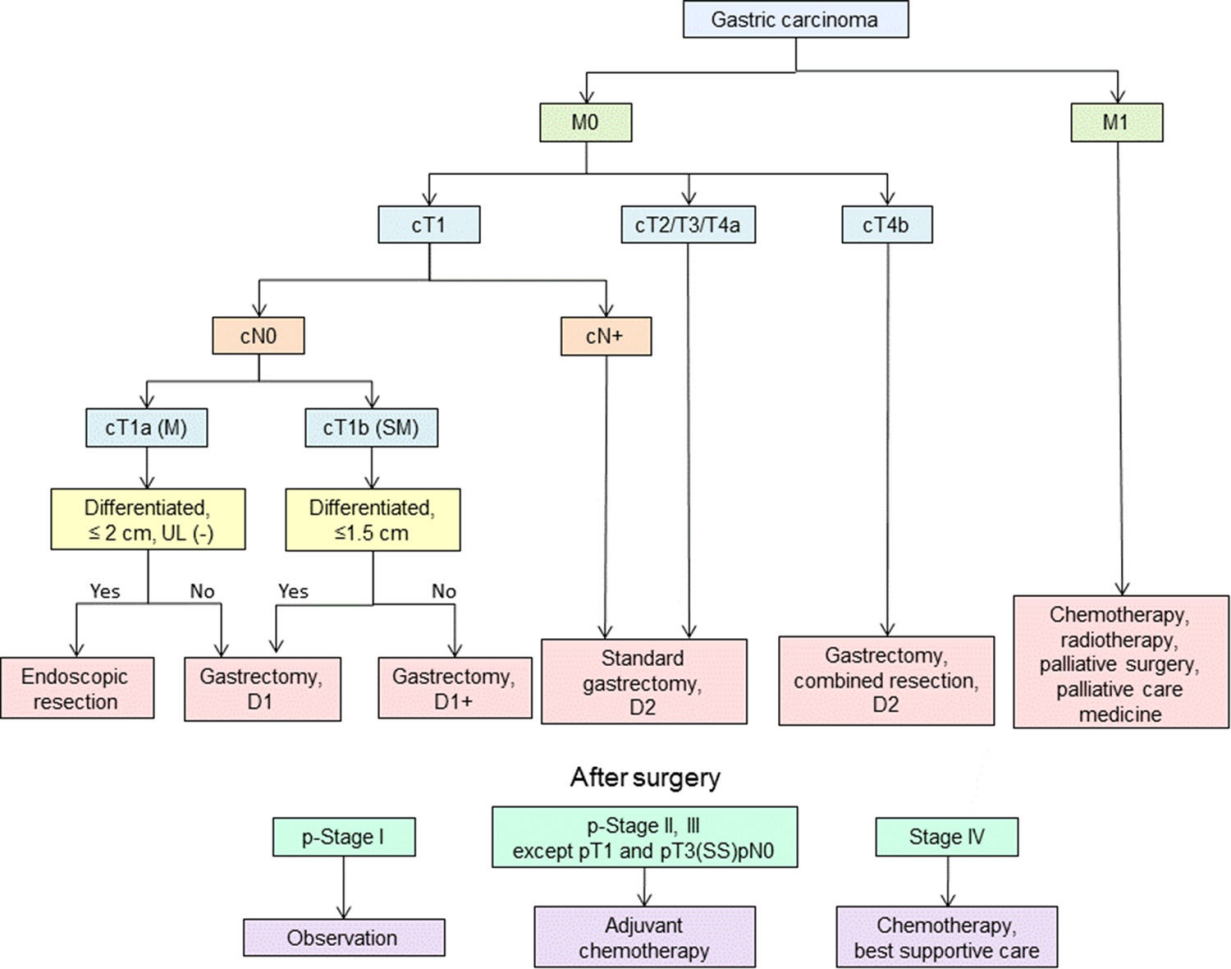
Algorithm of standard treatments

The algorithm is shown in Fig. 1.

### Investigational treatments

The following treatments show promise but are as yet to be established as the standard. They should be prospectively evaluated in appropriate clinical research settings. Patient consent for investigational treatments should be sought and the rationale behind them given.

The following constitute investigational treatments:

- Endoscopic submucosal dissection under the expanded criteria [see “Tumors indicated for endoscopic resection as an investigational treatment (expanded indication)”].
- Laparoscopic surgery for advanced cancer and those in need of total gastrectomy.
- Local tumor resection.
- Neoadjuvant chemotherapy.
- Adjuvant chemotherapy using agents other than S-1.
- Neoadjuvant chemoradiotherapy.
- Adjuvant chemoradiotherapy.

## Surgery

### Types and definitions of gastric surgery

#### Curative surgery

##### Standard gastrectomy

Standard gastrectomy is the principal surgical procedure performed with curative intent. It involves resection of at least two-thirds of the stomach with a D2 lymph node dissection.

##### Non-standard gastrectomy

In non-standard gastrectomy, the extent of gastric resection and/or lymphadenectomy is altered according to tumor stages.

*Modified surgery* The extent of gastric resection and/or lymphadenectomy is reduced (D1, D1+, etc.) compared to standard surgery.

*Extended surgery* (1) Gastrectomy with combined resection of adjacent involved organs. (2) Gastrectomy with extended lymphadenectomy exceeding D2.

#### Non-curative surgery

##### Palliative surgery

Serious symptoms such as bleeding or obstruction may develop in a patient with advanced/metastatic gastric cancer. Surgery to relieve symptoms may then be considered an option, and palliative gastrectomy or gastrojejunostomy is selected depending on the resectability of the primary tumor and/or surgical risks. Stomach-partitioning gastrojejunostomy has been reported to result in superior function compared to simple gastrojejunostomy [2].

##### Reduction surgery

The role of gastrectomy is unclear in patients with metastatic gastric cancer in the absence of urgent symptoms such as bleeding or obstruction. Reduction surgery aims to prolong survival or to delay the onset of symptoms by reducing tumor volume.

(*Additional comments in this English edition*) No evidence in support of reduction surgery was found in an international cooperative randomized controlled trial (REGATTA, JCOG0705/KGCA01 [3]).

### Extent of gastric resection

#### Surgery for gastric cancer

Surgery for gastric cancer is defined as follows in the order of the stomach volume to be resected.

- *Total gastrectomy* Total resection of the stomach including the cardia and pylorus.
- *Distal gastrectomy* Stomach resection including the pylorus. The cardia is preserved. In the standard gastrectomy, two-thirds of the stomach is resected.
- *Pylorus-preserving gastrectomy (PPG)* Stomach resection preserving the upper third of the stomach and the pylorus along with a portion of the antrum.
- *Proximal gastrectomy* Stomach resection including the cardia (esophagogastric junction). The pylorus is preserved.
- *Segmental gastrectomy* Circumferential resection of the stomach preserving the cardia and pylorus.
- Local resection.
- Non-resectional surgery (bypass surgery, gastrostomy, jejunostomy).

#### Determination of gastric resection

##### Resection margin

A sufficient resection margin should be ensured when determining the resection line in gastrectomy with curative intent. Proximal margin of at least 3 cm is recommended for T2 or deeper tumors with an expansive growth pattern (types 1 and 2) and 5 cm for those with an infiltrative growth pattern (types 3 and 4). When these rules cannot be observed, it is advisable to examine the proximal resection margin by frozen section. For tumors invading the esophagus, a 5-cm margin is not necessarily required, but frozen section examination of the resection line is desirable to ensure an R0 resection.

For T1 tumors, a gross resection margin of 2 cm should be obtained. When the tumor border is unclear, preoperative endoscopic marking by clips of the tumor border based on biopsy results will be helpful for decision making regarding the resection line.

##### Selection of gastrectomy

The standard surgical procedure for clinically node-positive (cN+) or T2-T4a tumors is either total or distal gastrectomy. Distal gastrectomy is selected when a satisfactory proximal resection margin (see above) can be obtained. Pancreatic invasion by tumor requiring pancreaticosplenectomy necessitates total gastrectomy regardless of the tumor location. Total gastrectomy with splenectomy should be considered for tumors that are located along the greater curvature and harbor metastasis to no. 4sb lymph nodes, even if the primary tumor could be removed by distal gastrectomy. For adenocarcinoma located on the proximal side of the esophagogastric junction, esophagectomy and proximal gastrectomy with gastric tube reconstruction should be considered, similarly to surgery for esophageal cancer.

For cT1cN0 tumors, the following types of gastric resection can be considered according to tumor location.

- Pylorus-preserving gastrectomy (PPG): for tumors in the middle portion of the stomach with the distal tumor border at least 4 cm proximal to the pylorus.
- Proximal gastrectomy: for proximal tumors where more than half of the distal stomach can be preserved.
- Segmental gastrectomy and local resection under sentinel navigation are still regarded as investigational treatments.

### Lymph node dissection

#### Extent of lymph node dissection

The extent of systematic lymphadenectomy is defined as follows according to the type of gastrectomy conducted. When the extent of lymphadenectomy performed does not fully comply with the D level criteria, the lymph node station that has been additionally resected or left in situ could be recorded as in the following examples: D1 (+No. 8a), D2 (−No. 10). However, when sending data to the nationwide database, the D level needs to be strictly decided upon and should be downgraded if resection of any of the lymph node stations that should have been resected was omitted.

##### Total gastrectomy (Fig. 2)

- D0: Lymphadenectomy less than D1.
- D1: Nos. 1–7.
- D1+: D1 + No. 8a, 9, 11p.
- D2: D1 + No. 8a, 9, 10, 11p, 11d, 12a.

For tumors invading the esophagus, D1+ includes:

No. 110*, D2 includes No. 19, 20, 110 and 111.

**Fig. 2:**
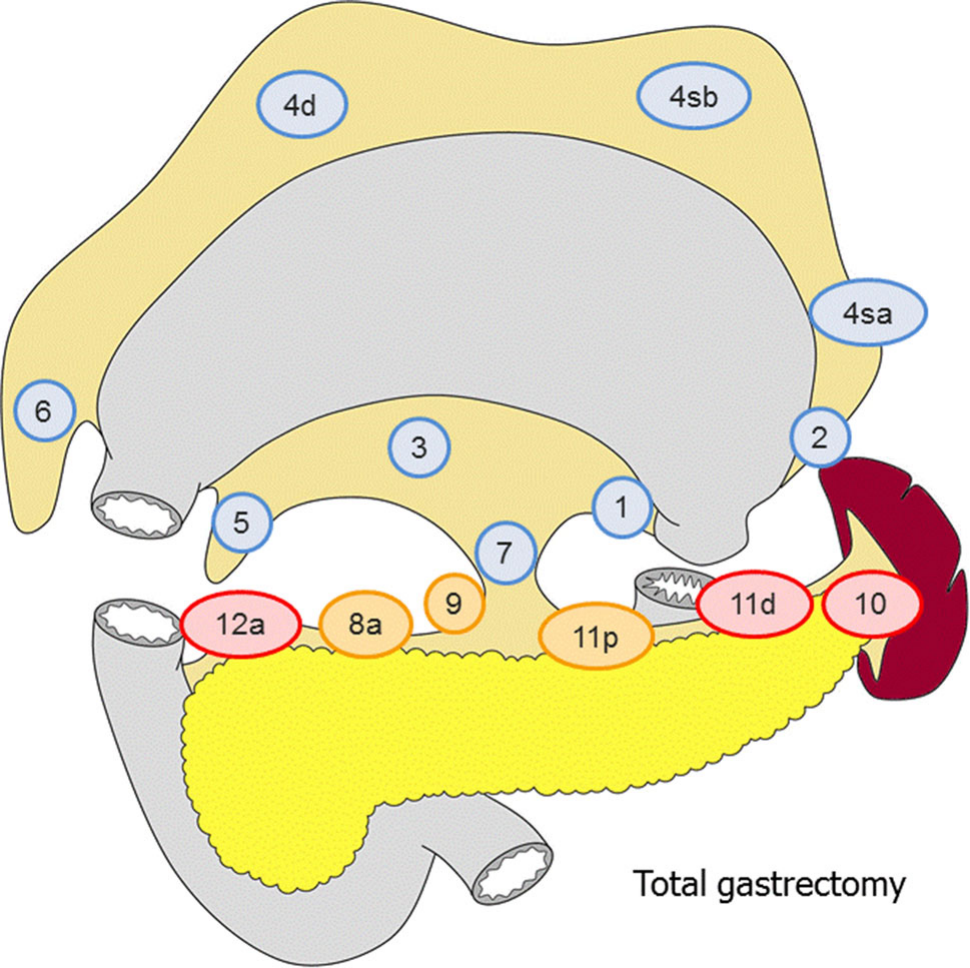
The extent of lymphadenectomy after total gastrectomy. The *numbers* correspond to the lymph node station as defined in the Japanese Classification of Gastric Carcinoma (1). Complete dissection of the nodes in* blue* denotes D1 dissection, the nodes in* orange* D1+ and the nodes in* red* D2

##### Distal gastrectomy (Fig. 3)

- D0: Lymphadenectomy less than D1.
- D1: No. 1, 3, 4sb, 4d, 5, 6, 7
- D1+: D1 + No. 8a, 9
- D2: D1 + No. 8a, 9, 11p, 12a.

**Fig. 3:**
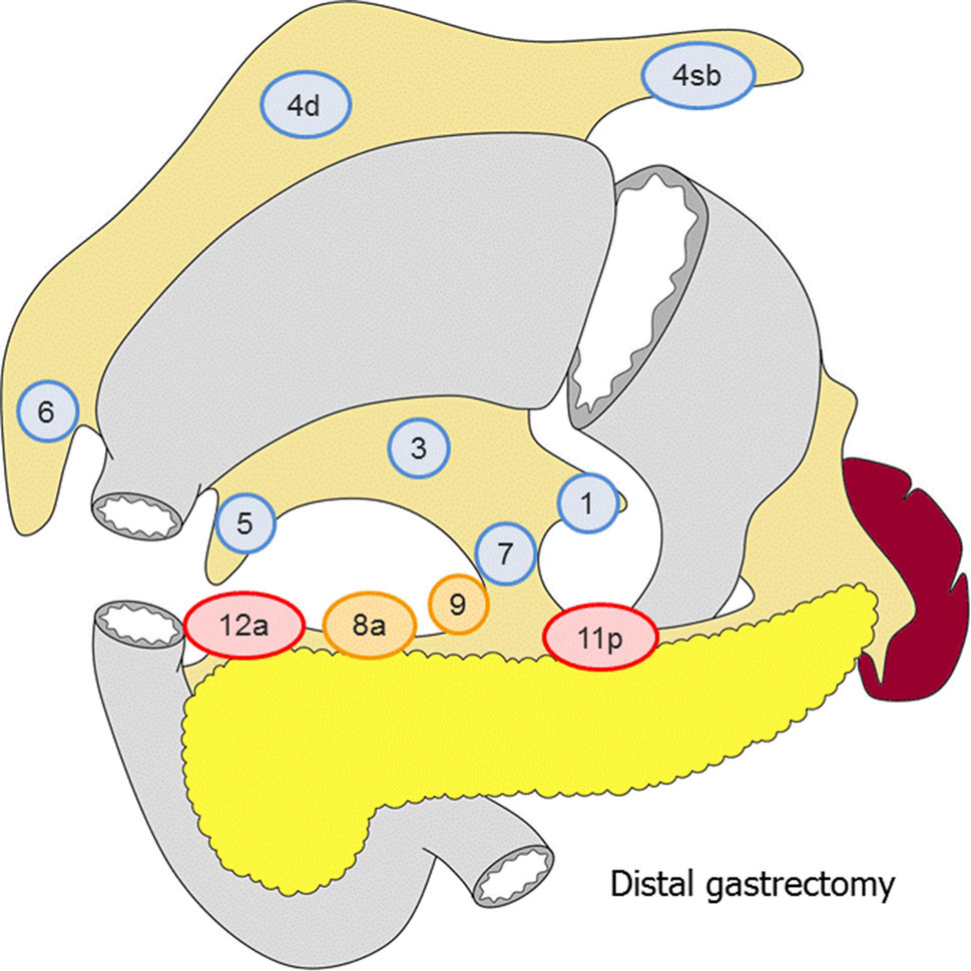
The extent of lymphadenectomy after distal gastrectomy. The *numbers* correspond to the lymph node station as defined in the Japanese Classification of Gastric Carcinoma (1). Complete dissection of the nodes in *blue* denotes D1 dissection, the nodes in* orange* D1+ and the nodes in* red* D2

##### Pylorus-preserving gastrectomy (Fig. 4)

- D0: Lymphadenectomy less than D1.
- D1: No. 1, 3, 4sb, 4d, 6, 7.
- D1+: D1 + No. 8a, 9.

**Fig. 4:**
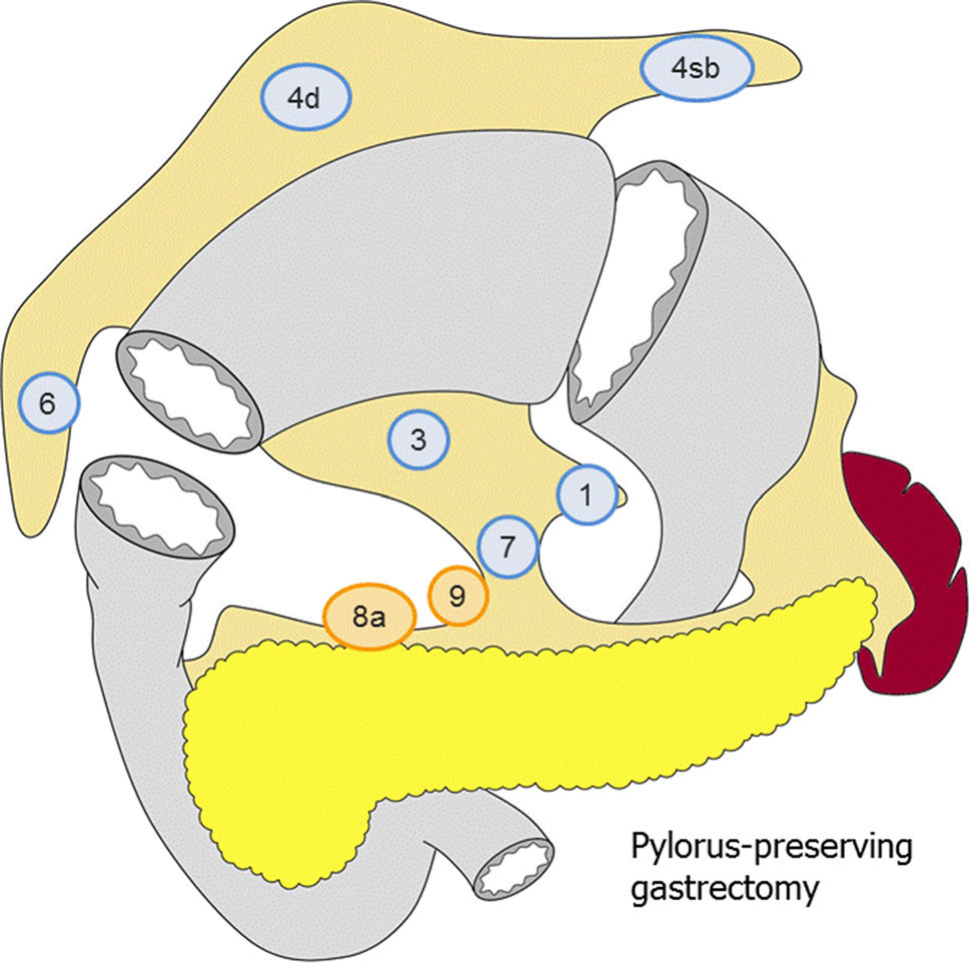
The extent of lymphadenectomy after pylorus-preserving gastrectomy. The *numbers* correspond to the lymph node station as defined in the Japanese Classification of Gastric Carcinoma (1). Complete dissection of the nodes in* blue* denotes D1 dissection and the nodes in* orange* D1+

##### Proximal gastrectomy (Fig. 5)

- D0: Lymphadenectomy less than D1.
- D1: No. 1, 2, 3a, 4sa, 4sb, 7.
- D1+: D1 + No. 8a, 9, 11p.

**Fig. 5:**
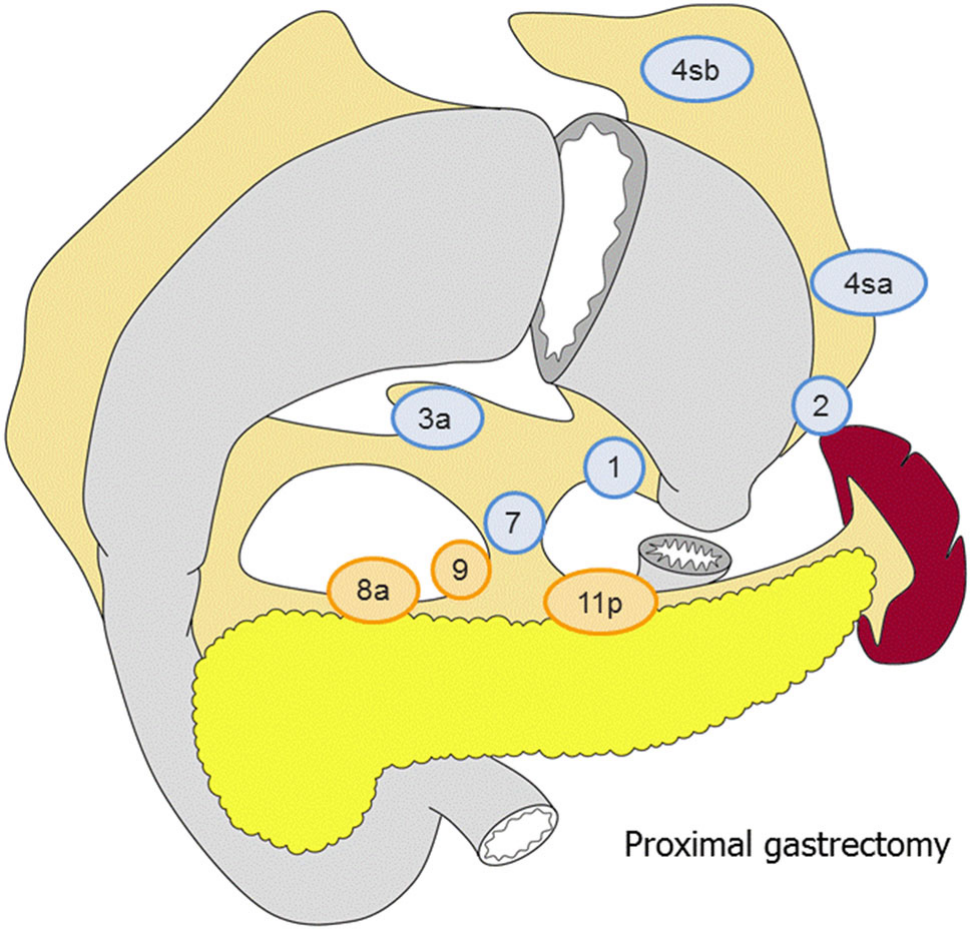
The extent of lymphadenectomy after proximal gastrectomy. The *numbers* correspond to the lymph node station as defined in the Japanese Classification of Gastric Carcinoma (1). Complete dissection of the nodes in* blue* denotes D1 dissection and the nodes in* orange* D1+

For tumors invading the esophagus, D1 + includes No. 110*.

*No. 110 lymph nodes (lower thoracic para-esophageal nodes) in gastric cancer invading the esophagus are those attached to the lower part of the esophagus that is removed to obtain a sufficient resection margin.

#### Indications for lymph node dissection

In principle, a D1 or a D1+ lymphadenectomy is indicated for cT1N0 tumors and D2 for cN+ or cT2-T4 tumors. Since the pre- and intraoperative diagnoses of lymph node metastases remain unreliable, a D2 lymphadenectomy should be performed whenever nodal involvement is suspected.

##### D1 lymphadenectomy

A D1 lymphadenectomy is indicated for T1a tumors that do not meet the criteria for EMR/ESD, and for cT1bN0 tumors that are histologically of differentiated type and 1.5 cm or smaller in diameter.

##### D1+ lymphadenectomy

A D1+ lymphadenectomy is indicated for cT1N0 tumors other than the above.

##### D2 lymphadenectomy

A D2 lymphadenectomy is indicated for potentially curable T2-T4 tumors as well as cT1N+ tumors. The role of splenectomy for complete resection of Nos. 10 and 11 nodes had long been an issue of controversy, and the final results of a randomized trial (JCOG 0110) are awaited. In the meantime, complete clearance of No. 10 nodes by splenectomy should be considered for potentially curable T2-T4 tumors invading the greater curvature of the upper stomach.

(*Additional comments in this English edition*) The randomized trial (JCOG 0110) was concluded and revealed non-inferiority of spleen preservation in terms of overall survival. Splenectomy should not be performed unless the primary T2-T4 tumor either directly invades the spleen or is located in the greater curvature of the upper stomach [4].

##### D2+ lymphadenectomy

Gastrectomy with extended lymphadenectomy beyond D2 is classified as a non-standard gastrectomy. Its role has been discussed as follows.

- The benefit of prophylactic para-aortic lymphadenectomy was denied by the randomized trial, JCOG 9501 [5].
- Although a R0 resection may be possible for tumors with para-aortic nodal involvement without other non-curative factors, the prognosis of this population is poor. Nevertheless, neoadjuvant chemotherapy followed by D2+ is a promising option (refer to CQ1).
- The role of No. 14v lymphadenectomy in distal gastric cancer is controversial. Dissection of No. 14v had been a part of D2 gastrectomy defined by the 13th edition of the Japanese Classification of Gastric Carcinoma, but was excluded from the previous version (version 3) of the Japanese Gastric Cancer Treatment Guidelines and remains that way in the current version. However, D2 (+No. 14v) may be beneficial for patients who are suspected to harbor metastasis to the No. 6 nodes.
- Involvement of No. 13 nodes is defined as M1 in the current version. However, D2 (+No. 13) lymphadenectomy may be an option in a potentially curative gastrectomy for tumors invading the duodenum [6].

### Junctional cancer

**Fig. 6:**
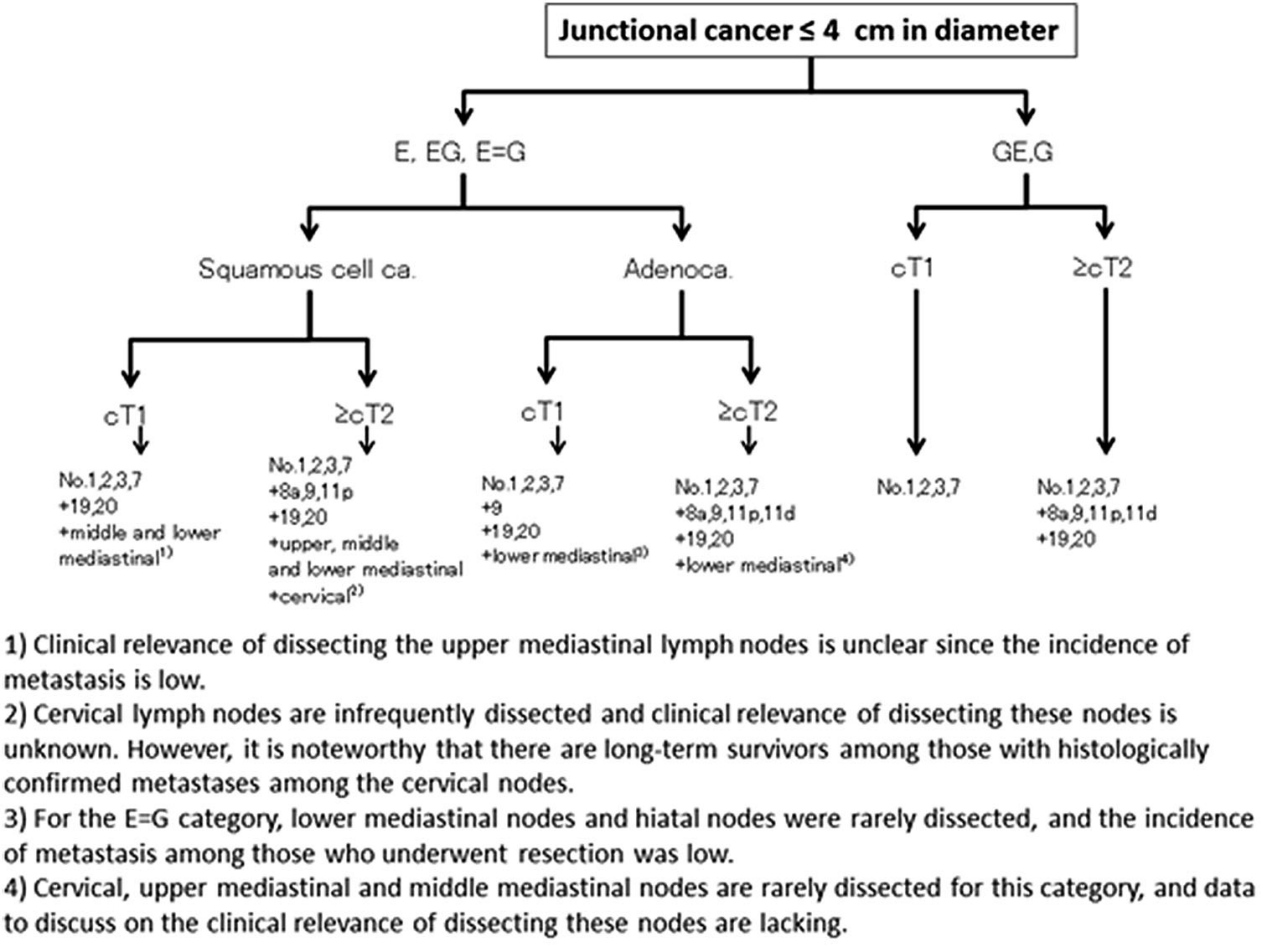
Algorithm showing the tentative standard in the extent of lymphadenectomy for junctional cancer based on the tumor location, histology and T-categories

In the Japanese Classification of Gastric Carcinoma, junctional cancer has been defined as cancer (adenocarcinoma or squamous cell carcinoma) with its center located within 2 cm of the esophago-gastric junction. There is no consensus over the type of resection and the extent of lymphadenectomy that could be a standard of care for this category. In 2012–2013, the Japanese Gastric Cancer Association and Japan Esophageal Society joined forces to conduct a nationwide surveillance of junctional cancer of ≤4 cm diameter, and retrospective data of 3177 patients operated on between 2001 and 2010 were collected from 273 institutions. An algorithm showing the tentative standard in the extent of lymphadenectomy based on the tumor location, histology and T-categories was constructed based on this surveillance (Fig. 6). The anatomical border between Nos. 19 and 20 and among Nos. 110, 111 and 112 cannot be defined clearly. Therefore, lower mediastinal nodes and hiatal nodes were each treated as one lymph node station in the current analysis. Dissection of No. 3b can be omitted when performing proximal gastrectomy. A prospective phase II study by the same joint force to further investigate this issue is on-going.

### Miscellaneous

#### Vagal nerve preservation

It is reported that preservation of the hepatic branch of the anterior vagus and/or the celiac branch of the posterior vagus contributes to improving postoperative quality of life through reducing post-gastrectomy gallstone formation, diarrhea and/or weight loss. In case of PPG, the hepatic branch should be preserved to maintain the pyloric function.

#### Omentectomy

Removal of the greater omentum is usually integrated in the standard gastrectomy for T3 (SS) or deeper tumors. For T1/T2 tumors, the omentum more than 3 cm away from the gastroepiploic arcade may be preserved.

#### Bursectomy

For tumors penetrating the serosa of the posterior gastric wall, bursectomy (removal of the inner peritoneal surface of the bursa omentalis) may be performed with the aim of removing microscopic tumor deposits in the lesser sac. There is no evidence that bursectomy reduces peritoneal or local recurrence, and it should be avoided in T1/T2 tumors to prevent injury to the pancreas and/or adjacent blood vessels.

A small-scale randomized controlled trial recently suggested a survival benefit for bursectomy in T3/T4a tumors [7]. A large-scale multi-institutional randomized trial to address this issue (JCOG 1001) was subsequently launched and has completed accrual.

#### Combined resection of adjacent organ(s)

For tumors in which the primary or metastatic lesion directly invades adjacent organs, combined resection of the involved organ may be performed in order to obtain an R0 resection.

#### Approaches to the lower esophagus

For gastric cancers invading less than 3 cm of the distal esophagus, a transhiatal abdominal approach is recommended [8]. Where a greater length of esophagus is involved, a transthoracic approach should be considered if the surgery is potentially curative.

#### Laparoscopic surgery

Laparoscopic surgery can be considered an option in general clinical practice to treat cStage I cancer that is indicated for distal gastrectomy. In the 2014 version of the guidelines by the Japan Society for Endoscopic Surgery, distal gastrectomy by the laparoscopic approach has been recommended for cStage I cancer (rated recommendation B). These decisions reflect the fact that the safety of the laparoscopic approach was proven in a prospective phase II study (JCOG0703) that involved only certified surgeons with sufficient experience [9] and that superiority in terms of short-term outcome has been reported through small-scale randomized trials and meta-analyses. However, surgeons will have to be aware that the learning-curve issue exists, and the indication for this approach should be decided discreetly in each institution based on the expertise of the staff members that participate in this type of surgery. Data regarding the long-term outcome are yet to be available, and results of pivotal phase III studies conducted in Japan (JCOG0912 [10]) and Korea (KLASS01 [11]) are awaited. As for more advanced cancer, there is currently no evidence to recommend a laparoscopic approach since randomized trials to look at safety and long-term outcome are currently ongoing (JLSSG0901, KLASS02).

Regarding total gastrectomy by this approach, no prospective trial has been reported. Thus, laparoscopic total gastrectomy has been rated by the guidelines of the Japan Society for Endoscopic Surgery (2014) as recommendation C1 (may be considered for a patient in need of total gastrectomy, but no scientific evidence in support of the procedure is currently available). Those who consider challenging the procedure should plan to do so with sufficient caution since postoperative complications were reported to be significantly more frequent in the first year of its introduction.

When conducting gastrectomy by the laparoscopic approach, informed consent should be obtained from all patients after providing sufficient information, including the lack of data regarding long-term consequences.

### Reconstruction after gastrectomy

The following reconstruction methods are usually employed. Each has advantages and disadvantages. Functional benefits of the pouch reconstruction are yet to be established.

#### Total gastrectomy

- Roux-en-Y esophagojejunostomy.
- Jejunal interposition.
- Double tract method.

#### Distal gastrectomy

- Billroth I gastroduodenostomy.
- Billroth II gastrojejunostomy.
- Roux-en-Y gastrojejunostomy.
- Jejunal interposition.

#### Pylorus-preserving gastrectomy

- Gastro-gastrostomy.

#### Proximal gastrectomy

- Esophagogastrostomy.
- Jejunal interposition.
- Double tract method.

## Endoscopic resection

### Methods of endoscopic resection

#### Endoscopic mucosal resection (EMR)

The lesion, together with the surrounding mucosa, is lifted by submucosal injection of saline (normo- or hypertonic) and removed using a high-frequency steel snare.

#### Endoscopic submucosal dissection (ESD)

The mucosa surrounding the lesion is circumferentially incised using a high-frequency electric knife (usually insulation-tipped), and the submucosal layer is dissected from the proper muscle layer.

### Handling of endoscopically resected specimens

#### Handling of resected specimens

The resected specimens should be handled according to the rules described in the Japanese Classification of Gastric Carcinoma [1].

#### Definition of differentiated-type and undifferentiated-type carcinoma

The tumor biopsy specimens and endoscopically resected tumors are histologically classified into either the differentiated or undifferentiated type. The former includes papillary adenocarcinoma (pap) and tubular adenocarcinoma (tub1, tub2), and the latter includes poorly differentiated adenocarcinoma (por1, por2) and signet-ring cell carcinoma (sig). Endoscopic dissection should be defined as non-curative if mucinous adenocarcinoma (muc) was found in the submucosal layer, regardless of whether it is considered to derive from the differentiated or undifferentiated type.

#### Histological predominance and intratumoral ulcerative findings (UL)

A tumor consisting of components of both differentiated- and undifferentiated-type carcinoma is nevertheless classified into one of the two types according to the quantitative predominance. In addition, when more than one histological type is found in a tumor, all histological types are to be recorded in the order of quantitative predominance, e.g., tub2 > tub1. Diagnosis of UL(+) is principally made based on the histological evidence of ulcerative findings. However, endoscopic and/or radiological evidence should also be taken into consideration when making a conclusive diagnosis. A biopsy-derived scar is usually observed histologically as fibrosis restricted to small areas just beneath the muscularis mucosae. However, if it cannot be discriminated from the ulcer scar, it should be classified as UL(+).

### Indication for endoscopic resection (Fig. 7)

#### Principles of indication

Endoscopic resection is considered for tumors that have a very low possibility of lymph node metastasis and are suitable for en-bloc resection.

**Fig. 7:**
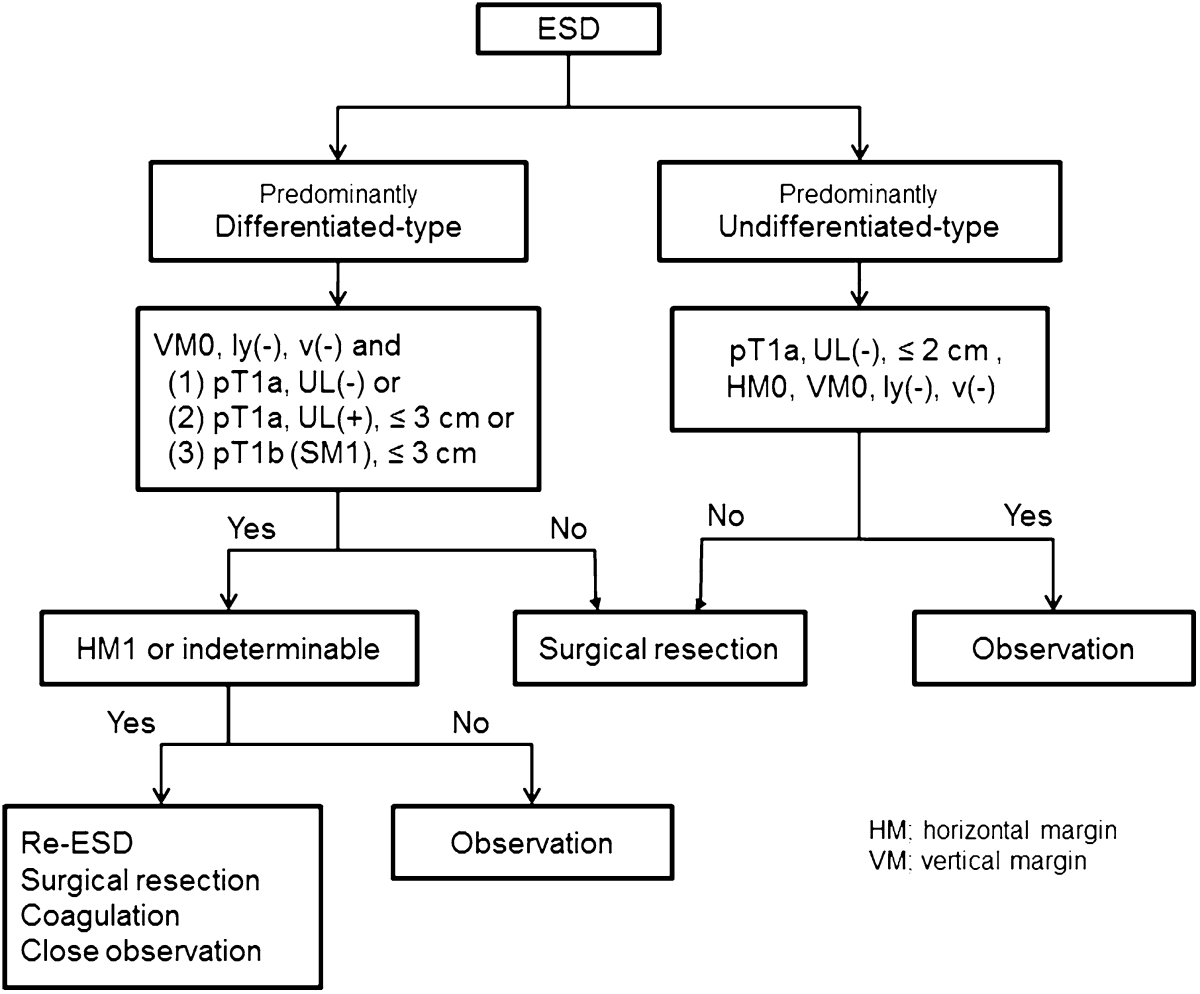
Algorithm showing treatment of early gastric cancer according to the histopathologic findings of the specimens resected by ESD

Since compilation of the first version of this guideline, two independent sets of indications for the endoscopic resection have been provided: an absolute indication for standard EMR/ESD and an expanded indication for ESD to be considered as an investigational treatment. The evidence regarding curability of the latter technique remains insufficient, and the procedure should be offered with caution.

#### Tumors indicated for endoscopic resection as a standard treatment (absolute indication)

EMR or ESD is indicated as a standard treatment for the following tumor.

- A differentiated-type adenocarcinoma without ulcerative findings [UL(−)], of which the depth of invasion is clinically diagnosed as T1a and the diameter is ≤2 cm.

#### Tumors indicated for endoscopic resection as an investigational treatment (expanded indication)

Tumors of the following categories have very low possibility of lymph node metastasis when they are not accompanied with lymphovascular infiltration [ly(−), v(−)] and could be indicated for endoscopic resection [12, 13]. To avoid incomplete dissection, ESD rather than EMR should be performed. Since evidence on long-term survival is currently lacking, however, endoscopic treatment for these lesions should be considered investigational until trials such as JCOG0607 turn out to be positive.

Tumors clinically diagnosed as T1a and

- Of differentiated-type, UL(−), but >2 cm in diameter.
- Of differentiated-type, UL(+), and ≤3 cm in diameter.
- Of undifferentiated-type, UL(−), and ≤2 cm in diameter.

#### Local recurrence after EMR/ESD

Local mucosal recurrence after EMR/ESD for tumors that had fulfilled the absolute indication could be considered to meet the criteria for expanded indication and may be treated by another ESD. However, given paucity of the evidence in terms of long-term survival, repeat ESD should be also considered as investigational.

### Curability of endoscopic resection

Meticulous pathologic examination of the resected specimen is mandatory. Curability needs to be assessed based on both the results of the pathologic examination and facts based on the accumulated data. Two factors should be considered for curability assessment: completeness of the primary tumor removal and nil possibility of lymph node metastasis.

#### Curative resection

The resection is determined as curative when all of the following conditions are fulfilled: en bloc resection, tumor size ≤2 cm, histologically of differentiated type, pT1a, negative horizontal margin (HM0), negative vertical margin (VM0) and no lymphovascular infiltration (ly(−), v(−)).

#### Curative resection for tumors of expanded indication

The resection is considered as curative when all of the following conditions are fulfilled:

En bloc resection, HM0, VM0, ly(−), v(−), and

- Tumor size >2 cm, histologically of differentiated type, pT1a, UL(−).
- Tumor size ≤3 cm, histologically of differentiated type, pT1a, UL(+).
- Tumor size ≤2 cm, histologically of undifferentiated type, pT1a, UL(−).
- Tumor size ≤3 cm, histologically of differentiated type, pT1b (SM1, <500 micron from the muscularis mucosae).

As the evidence is still insufficient for differentiated type tumors accompanied with some areas of undifferentiated type histology, the following resections are regarded as non-curative for the time being, and addition of surgical treatments should be recommended.

- Areas of undifferentiated type carcinoma exceed 2 cm in the above (a).
- Undifferentiated type component in the part that had invaded the submucosa in the above (d).

A new rule in the current version is that if a component of undifferentiated type carcinoma was found in the above (b) but was not the predominant histological type, risk of nodal metastasis is estimated to be low [14], and the endoscopic resection will be regarded as curative.

#### Non-curative resection

Resection that does not satisfy any of the above criteria is considered non-curative.

### Treatments after endoscopic resection

#### Treatments after curative resection

Follow-up with annual or biannual endoscopy is recommended.

#### Treatments after curative resection for tumors of expanded indications

Follow-up with abdominal ultrasonography or CT scan as well as annual or biannual endoscopy is recommended.

In case of either “Treatments after curative resection” or “Treatments after curative resection for tumors of expanded indications,” it has been recommended that *Helicobacter pylori* be examined and, if positive, be eradicated. However, some studies showed that Helicobacter eradication after ER had no impact on the occurrence of metachronous cancer. Further investigations regarding this issue are warranted.

#### Treatment after non-curative resection

Surgical treatment should be performed after non-curative resection. However, as the following cases actually carry very low risk for harboring lymph node metastasis, non-surgical treatments such as repeated ESD, endoscopic coagulation using a laser or argon-plasma coagulator, or close observation expecting a burn effect of the initial ESD could be proposed as alternatives and delivered upon the patient’s informed consent.

- En bloc resection of a differentiated type carcinoma with positive horizontal margin (HM1) as the only non-curative factor.
- Piecemeal resection of a differentiated type carcinoma satisfying all other criteria.

When these cases come from the category (b) or (d) of the “Curative resection for tumors of expanded indication,” the size of the residual mucosal lesion should be re-assessed by endoscopy. If the sum of the lengths of the resected and residual lesions exceeds 3 cm, surgery is indicated. When the positive horizontal margin or the piecemeal resection margin involves the part of submucosal invasion in category (d), surgery is indicated.

## Chemotherapy

Although recent advances in chemotherapy have achieved considerable tumor regression in many cases of unresectable/recurrent gastric cancer, these responses have not ultimately led to complete cure. The median survival time achieved in clinical trials for the disease at this stage remains 6–13 months. The current goal of chemotherapy therefore is to delay the manifestation of disease-related symptoms and/or to prolong survival. Survival benefit of chemotherapy has been proven in randomized controlled trials comparing chemotherapy with best supportive care in patients with unresectable gastric cancer with performance status (PS) of 0–2. Although very rare, some patients with advanced disease even survive more than 5 years by chemotherapy alone. Thus, chemotherapy is the treatment to be primarily considered for unresectable/recurrent gastric cancer among patients with sufficiently good PS.

### Principles of indication

Chemotherapy is indicated for patients with unresectable or recurrent disease, or those after non-curative R2 resection, whose general condition and major organ functions are preserved: to be specific, patients of PS 0–2, with unresectable T4b disease, extensive nodal disease, hepatic metastases, peritoneal dissemination or other M1 disease.

### Recommendable regimens for Japanese patients

Treatment regimens were classified into the following three categories according to the degree that the regimen could be recommended. The recommendation category was determined by the committee members based on the levels of reported evidence and, ultimately, through consensus reached after thorough literature review and discussion among the members.

- Recommendation category 1: treatment regimens that are recommended in clinical practice.
  Regimens included in this category will need to be either superior or non-inferior to the standard treatment in a phase III trial with overall survival as the primary endpoint. In addition, consensus must be reached within the committee members on the interpretation of the phase III study results, availability of the drugs in Japan and sufficient safety and efficacy data with the Japanese participants.
- Recommendation category 2: treatment regimens that could be selected in clinical practice.
  Regimens in this category include the following: (1) those that were found to be superior or non-inferior to the standard treatment in a phase III trial but failed to gain sufficient support from the committee members to be included in category 1; (2) those with sufficient efficacy and safety data obtained in a phase II trial and consensus reached among the committee members.
- Recommendation category 3: treatment regimens that cannot be recommended in clinical practice.
  Regimens in this category either failed to show superiority or non-inferiority in terms of overall survival in a phase III trial or were lacking in sufficient efficacy/safety data with the Japanese participants.

#### First-line treatment

##### HER2 testing

Since a trastuzumab-containing regimen became the standard of care for HER2-positive gastric cancer, HER2 testing is strongly recommended in all patients who will undergo chemotherapy for unresectable/metastatic gastric cancer.

##### HER2-negative gastric cancer

S-1 + cisplatin combination is the standard of care (recommendation category 1) based on the results of two phase III trials conducted in Japan (SPIRITS trial [15] and JCOG 9912 trial [16]).

Capecitabine + cisplatin combination is currently one of standard treatments overseas and was employed as a control group in global phase III studies, the ToGA trial [17] and AVAGAST trial [18]. Since subset analyses of the Japanese participants in these trials have shown safety and efficacy, this combination can be selected in clinical practice (recommendation category 2).

S-1 + docetaxel combination failed to show superiority to S-1 monotherapy in the primary survival analysis of the START trial conducted with the Japanese and Korean participants, but superiority in overall survival was observed in a reanalysis after clarifying outcome of several censored cases [19]. This regimen could be selected for a limited population such as those who wish to be treated at the outpatient clinic (recommendation category 2).

Irinotecan + cisplatin and S-1 + irinotecan combinations are not recommended as the first-line regimen because they did not show significant superiority over 5-FU alone and S-1 alone, respectively, in the randomized trials conducted in Japan [16, 20] (recommendation category 3).

Regarding triplet regimens, efficacy of infusional 5FU + cisplatin + docetaxel was proven in the V325 study [21] conducted in the western countries. Given the excessive toxicity and lack of data for Japanese patients, this regimen cannot be recommended for general practice (recommendation category 3). In Japan, a triplet consisting of S-1, cisplatin and docetaxel (DCS regimen) is currently being evaluated in a phase III trial, JCOG1013, following some phase II results. Thus, the DCS regimen currently needs to be regarded as an investigational treatment.

Evidence is lacking regarding chemotherapy for specific types of patients such as those with no oral intake, peritoneal carcinomatosis (patients with a moderate to high volume of ascites or bowel obstruction) and the elderly (refer to CQ5 and CQ6).

(*Additional comments in this English edition*) Following the results of the SOX-GC trial [22] and REAL2 trial [23], S-1 plus oxaliplatin or capecitabine plus oxaliplatin became options for the first-line chemotherapy and is tentatively rated as recommendation category 2.

##### HER2-positive gastric cancer

IHC3+ or FISH positive patients were eligible for the ToGA trial [17]. In the subgroup analyses of the trial, survival benefit was more distinct when IHC3+ or FISH positive/IHC2+ cohorts were selected. Thus, trastuzumab-containing regimens will be recommended for patients with IHC3+ or FISH positive/IHC2+ status. Following the results of the ToGA trial, a combination of trastuzumab, cisplatin and either capecitabine or infusional 5FU will be recommended for clinical practice (recommendation category 1). A phase II trial to explore trastuzumab combined with triweekly S-1 + cisplatin was conducted with promising results. However, this combination will remain in category 2 at this time because of the small volume of efficacy and safety data.

#### Second-line treatment

Second-line treatment is recommended for patients with sufficient performance status, following several randomized trials mentioned below. Monotherapy with any of the three agents, docetaxel, irinotecan or paclitaxel (weekly administration), can be recommended (recommendation category 1).

Randomized trials conducted in Germany [24] and Korea [25] showed a significant survival advantage of the second-line treatment (docetaxel or irinotecan) over the best supportive care. A Japanese phase III trial, WJOG4007 [26], failed to prove superiority in overall survival of irinotecan over paclitaxel (weekly administration), but did show median survival time for both groups of approximately 9 months, which is favorable when compared with survival data from other trials exploring the second-line chemotherapy. This could be explained by the fact that a high proportion of participants in this trial received another line of treatment (taxanes among patients allocated to the irinotecan group and irinotecan among those allocated to the paclitaxel group). Thus, this trial indicates that patients with good performance status could benefit from the third-line treatments.

(*Additional comments in this English edition*) Following the results of the RAINBOW trial [27] and the REGARD trial [28], paclitaxel + ramucirumab emerged as a new standard of care and was rated as recommendation category 1 in the second-line setting, while ramucirumab monotherapy was rated as recommendation category 2 alongside monotherapies with docetaxel, irinotecan or paclitaxel, which were relegated from recommendation category 1.

### Chemotherapy as a general practice

#### Indication

Indication for chemotherapy should be decided after taking into consideration the following eligibility criteria.

1. Clinical and pathological diagnosis of gastric cancer has been obtained.
2. PS of 0–2. Generally speaking, chemotherapy is not indicated for patients with PS of 3 or 4. Indication for chemotherapy should be discreetly decided considering the toxicity and benefit. The safety issue may be of particular concern when a patient suffers from massive ascites or overt peritoneal carcinomatosis.
3. Major organ functions are preserved.
4. Patient does not suffer from severe comorbidities.
5. Informed consent has been obtained from the patient.

#### Methodology

1. Prior to treatment, PS, body weight, clinical symptoms and laboratory data (including examination for hepatitis virus) should be checked, and imaging studies such as computerized tomography (CT) should be performed to obtain baseline measurements of the lesions.
2. Response to the treatment should be evaluated by examinations that may include CT, endoscopy and contrast radiography, followed by comparison with the baseline data. Tumor shrinkage should be evaluated by response criteria of the Japanese Classification of Gastric Carcinoma or Response Evaluation Criteria in Solid Tumors (RECIST) to decide on whether or not to continue with the treatment.
3. When continuation of the treatment is deemed oncologically feasible, the drug dosage and administration schedule should be reconsidered taking into account the adverse events observed in the previous cycle of treatment. Attention should also be paid to cumulative adverse events such as skin manifestations, taste disturbance and neurotoxicity.
4. Chemotherapy for individuals exposed or infected to hepatitis B virus should be screened, monitored and treated by referring to “Chapter 6.3., HBV reactivation” of the Japan Society of Hepatology Guidelines for the Management of Hepatitis B Virus Infection to prevent reactivation.

#### Drugs to be used

The following drugs are used in chemotherapy for gastric cancer: fluorouracil (5FU), tegafur-gimestat-otastat potassium (S-1), capecitabine, cisplatin, irinotecan, docetaxel, paclitaxel and trastuzumab. These drugs are to be used alone or in combination, adhering to the dose and schedule employed when being evaluated in clinical trials.

(*Additional comments in this English edition*) Ramucirumab and oxaliplatin can now be added to the list of the drugs.

### Postoperative adjuvant chemotherapy

Postoperative adjuvant chemotherapy is delivered with an intention to reduce recurrence by controlling residual tumor cells following curative resection. Various regimens had been tested in numerous clinical trials in Japan without producing solid evidence in support of adjuvant chemotherapy until the efficacy of S-1 was proven in the ACTS-GC trial [29, 30], a study that secured the place of postoperative chemotherapy with S-1 as a standard of care (recommendation category 1). After this, the feasibility of several combinations of anticancer drug with S-1 was explored in the postoperative setting [31, 32], and some of the combinations are currently under evaluation in phase III trials. On the other hand, other phase III evidence in support of postoperative chemotherapy was established in 2012 by the CLASSIC trial conducted mainly in Korea [33], in which significant prolongation of recurrence-free survival was shown with a combination of capecitabine and oxaliplatin. However, oxaliplatin has not been approved for gastric cancer in Japan as of 2014. Survival benefit of postoperative adjuvant chemotherapy by combination of S-1 and another cytotoxic drug, including oxaliplatin, will have to be proven by a randomized trial with S-1 monotherapy as a control.

(*Additional comments in this English edition*) Following the results of J-CLASSIC [34] and SOX-adjuvant trials [35], capecitabine or S-1 plus oxaliplatin has been approved as an adjuvant regimen in Japan.

#### Indications

The patients eligible for the ACTS-GC trial were those with a tumor of pathological stage II, IIIA or IIIB, excluding those classified as stage II due to pT1/pN2·pN3 status, as defined by the previous 13th edition of the Japanese Classification of Gastric Carcinoma (2nd English edition), who had undergone R0 gastrectomy with ≥D2 lymphadenectomy. The eligibility for postoperative adjuvant chemotherapy will remain the same in the current version of the treatment guidelines.

However, in the 14th edition of the Japanese Classification of Gastric Carcinoma (3rd English edition) whose staging scheme is the same as the 7th edition of UICC/TNM, stage IIA includes pT3(SS)/N0 status, which had been rated as stage IB in the 13th edition and therefore was ineligible for the ACTS-GC. In other words, the eligibility criteria will remain unchanged by excluding this population as well as the pT1/pN2·pN3 population from stage II/III.

Additionally, what has been written in “Chemotherapy as a general practice” applies also to chemotherapy in the adjuvant setting except that response to the treatment cannot be evaluated by imaging modalities until disease recurrence.

#### Administration schedule

S-1 is to be started within 6 weeks from surgery, after sufficient recovery from the surgical intervention. A 6-week cycle consisting of 4 weeks of daily oral administration of S-1 at a dose of 80 mg/m^2^ followed by 2 weeks of rest is repeated during the 12 months after surgery (8 cycles). Since postoperative patients are generally more vulnerable to both hematological and non-hematological adverse events, appropriate dose reduction and schedule modification should be considered, including a switch to a schedule of 2 weeks of administration followed by 1 week of rest.

## Palliative care

Palliative care is an approach that improves the quality of life of patients and their families facing the problems associated with life-threatening illness through the prevention and relief of suffering by means of early identification and impeccable assessment and treatment of pain and other problems, physical, psychosocial and spiritual (WHO Definition of Palliative Care, 2002). The importance of palliative care increases incrementally as cancer progresses. The knowledge and technique to cope with pain, to communicate and to manage symptoms are required. Methods to accomplish these aims include radiotherapy and psychotherapy in addition to medication. Various clinical studies are on-going with particular emphasis on pain control.

## Clinical pathway after surgery for gastric cancer

It is extremely difficult to establish a clinical pathway for patients undergoing gastric cancer surgery that is widely applicable to various surgical procedures and institutions. However, it is possible to propose some core items based on which individual pathways could be constructed, and these could contribute to reducing disparities in surgical management for gastric cancer. A basic pathway has been constructed around the timing of some core items such as removal of the nasogastric tube, initiation of oral fluid intake, initiation of solid food intake, administration of antibiotics, stoppage of intravenous fluid administration and discharge from the hospital (Table 1). This clinical pathway is applicable to all surgical procedures including distal, total and proximal gastrectomy regardless of whether the surgery was performed laparoscopically or by open approach. However, postoperative management should be individualized for high-risk patients with severe comorbidities that include impaired cardiac, pulmonary, hepatic or renal functions. Recently, investigators have been inclined to aim for further shortening of postoperative hospital stays through the concept of ERAS (enhanced recovery after surgery), but the value of such programs in gastric cancer surgery is yet to be defined.

**Table 1:** A common clinical pathway for distal, total and proximal gastrectomy

Clinical items	Date on the clinical pathway
Removal of nasogastric tube	Before or on postoperative day 1
Initiation of oral fluid intake	On or after postoperative day 1
Initiation of solid food intake	Between postoperative days 2–4
Prophylactic administration of antibiotics	Only on the day of operation
Removal of epidural tube	Before or on postoperative day 3
Removal of urinary catheter	Before or on postoperative day 3
Intravenous fluid administration	Until postoperative day 5–7
Removal of intra-abdominal drains	Before or on postoperative day 5
Discharge from the hospital	Between postoperative days 8–14

## Follow-up surveillance after surgery for gastric cancer

Follow-up at the outpatient clinic could be helpful so that the patients can readjust to their lives at home, cope with postgastrectomy symptoms and overcome the nutritional issues. In addition, surveillance for early detection of recurrence and secondary cancer is usually conducted according to the level of risk for recurrence, estimated based on the clinical stages. However, evidence that such surveillance actually improves survival is lacking. Due to the paucity of prospective studies that explored follow-up programs after gastrectomy, it is not possible to make any recommendation on how often the examinations should be performed, or even on which examination to perform. However, some retrospective studies suggest that CT, measurement of tumor markers (CEA and CA19-9) and endoscopy are effective to detect recurrence, gastric remnant cancer and metachronous multiple cancer. Tumor markers, when applicable, are apt to rise 2–3 months before metastatic lesions become detectable by imaging modalities. Models of follow-up programs for early-stage cancer and advanced cancer are shown in Tables 2 and 3.

**Table 2:** Postoperative follow-up for stage I gastric cancer patients

Duration after surgery	Year:			1	1.5	2	2.5	3	4	5
	Month:	1	6	12						
Medical examination, PS, body weight		○	○	○	○	○	○	○	○	○
Blood test including tumor markers		○	○	○	○	○	○	○	○	○
CT and/or US			○	○		○		○	○	○
Endoscopy				○				○		○

Examinations to be considered when needs arise: chest X-ray gastrography, barium enema, colonoscopy, bone scintigram, PET scan

Other surveillance programs should be sought beyond the 5th year

**Table 3:** Postoperative follow-up for stage II–III gastric cancer patients

Duration after surgery	Year:					1				2	2.5	3	3.5	4	4.5	5
	Month:	1	3	6	9	12	15	18	21	24						
Medical examination, PS, body weight		○	○	○	○	○	○	○	○	○	○	○	○	○	○	○
Blood test including tumor markers		○	○	○	○	○	○	○	○	○	○	○	○	○	○	○
CT and/or US				○		○		○		○	○	○		○		○
Endoscopy						○						○				○

Examinations to be considered when needs arise: chest X-ray, gastrography, barium enema, colonoscopy bone scintigram, PET scan

Other surveillance programs should be sought beyond the 5th year

Follow-up should continue for no longer than 5 years after which patients should be referred to regional general physicians or should be encouraged to undergo surveillance examinations provided as a part of health care programs in their districts or at their places of work. In that aspect, collaboration among various levels of medical facilities is needed to provide comprehensive care to gastric cancer survivors. Ultimately, there remains a need to scientifically verify the prognostic relevance of postoperative follow-up programs.

## Appendix: clinical questions

*CQ1*. Should surgery be avoided in patients with gastric cancer when metastasis to the para-aortic lymph nodes has been detected?

*Answer* A multidisciplinary approach including surgery with para-aortic lymph node dissection could be proposed when para-aortic lymph node metastases are confined to the No. 16 a2–b1 region, provided other non-curative factors are absent.

Para-aortic metastases from gastric cancer are classified as M1, and surgery with curative intent is not indicated according to the treatment algorithm of the current guidelines (refer to “Algorithm of standard treatments to be recommended in clinical practice”).

Systemic para-aortic lymph node dissection (PAND) had been attempted in Japan as clinical studies until its survival benefit was denied in a randomized trial in which only patients without lymphadenopathy in the para-aortic region were eligible [5]. However, numerous retrospective data from patients who underwent PAND are available in Japan and these almost invariably indicate that (1) metastases to the para-aortic nodes are pathologically confirmed in a certain percentage of these patients and (2) cure was achieved in approximately 10–20 % of the patients who harbored metastases to the para-aortic nodes. A similar result was recently reported from a Western country [36]. Thus, it is not possible to totally deny the survival benefit of PAND when lymphadenopathy restricted to the No. 16 a2–b1 region is found by preoperative imaging studies.

As a multidisciplinary treatment for this population, a treatment strategy of two courses of neoadjuvant chemotherapy with S-1 + cisplatin followed by PAND was explored in a phase II trial. Patients with bulky nodal disease with or without lymphadenopathy restricted to the No. 16 a2–b1 region were eligible, and peritoneal metastasis as well as the CY1 status had to be ruled out by staging laparoscopy prior to registration. Since a 5-year survival rate of 53 % was reported in this trial [37], this treatment strategy could be recommended for institutions with sufficient expertise in PAND. On the other hand, there are arguments that a 5-year survival rate of around 10 % can be achieved by chemotherapy when the para-aortic lymph node metastasis is the only factor that renders patients incurable [38, 39]. However, these retrospective studies are known to include patients who eventually underwent surgery after responding to the chemotherapy and may at least partially reflect the benefit of the multidisciplinary approach.

*CQ2*. How should gastric cancer with hepatic metastases be treated?

*Answer* A multidisciplinary approach including surgery with curative intent could be proposed when the number of metastatic nodules is small, provided other non-curative factors are absent.

Hepatic metastases from gastric cancer are classified as M1, and surgery with curative intent is not indicated according to the treatment algorithm of the current guidelines (refer to “Algorithm of standard treatments to be recommended in clinical practice”).

Hepatic metastases from gastric cancer are often deemed unresectable since they are liable to be found as multiple nodules distributed to both hepatic lobes and are likely to be accompanied with metastatic lesions outside of the liver. No prospective trial exploring a benefit of hepatectomy has been conducted, and only retrospective analyses of small cohorts collected over several decades mostly as single-institution studies [40–42] are available. However, 5-year survival rates ranging from 10 to 40 % have been reported from these studies, and one cannot deny a possibility that hepatectomy results in long-term survival among highly selected patients. Solitary metastasis or a small number of metastatic nodules has been highlighted as a favorable prognostic factor in most of the studies [43]. Given the recent advances in imaging studies, and the fact the diagnosis of solitary metastasis could be unreliable in older cases, hepatectomy may be considered for patients with a small number of metastatic nodules, and not restricted to a solitary tumor, provided that there is no other non-curative factor. Since there was no agreement on whether the synchronous metastases fare better than metachronous metastases, surgery could also be considered for recurrences in the liver if they fulfill the conditions mentioned above. Most patients eventually suffer from recurrences, however, and perioperative chemotherapy could be recommended for the population that had not been treated by adjuvant chemotherapy prior to detection of the hepatic metastases. Evidence on which chemotherapeutic regimen can be recommended in this particular setting, however, is totally lacking.

*CQ3*. How should gastric cancer with positive peritoneal cytology (CY1) be treated? Could there be any therapeutic proposal for patients who underwent gastrectomy and were found afterwards to have been CY1 (in some institutions, results of the cytologic examination are available only after surgery in case the sample was collected at surgery)?

*Answer* Multidisciplinary treatment including standard gastrectomy can be proposed for patients with no other non-curative factors. If the CY1 status was revealed after surgery, postoperative treatment with S-1 can be recommended as the tentative standard.

In Japan, peritoneal washing samples are usually collected during surgery for cytologic examination to detect free cancer cells. Free cancer cells in the peritoneal cavity (CY1) are classified as M1, and surgery with curative intent is not indicated according to the treatment algorithm of the current guidelines (refer to “Algorithm of standard treatments to be recommended in clinical practice”). However, patients with CY1 status are often treated by standard gastrectomy in the absence of other no-curative factors. The outcome of these patients had originally been dismal with a median survival time of approximately 12 months and 5-year survival rate of 7.8 %, but such data often included patients who were treated with surgery alone [44].

More recently, a prospective phase II study was conducted in which technically resectable cancer with CY1 as the only non-curative factor (patients with minimal and resectable peritoneal deposits included) was treated by standard gastrectomy followed by S-1 monotherapy until disease progression. The median recurrence-free and overall survival time in this study were 376 and 705 days, and 5-year recurrence-free and overall survival rates were 21 and 26 %, respectively [45]. In addition, a single-institution retrospective study of 120 CY1 patients who underwent surgery followed by S-1 monotherapy revealed a 5-year survival rate of 26.6 % [46], which was compatible with the trial result. These results are far better than the results obtained before S-1 became available and are equivalent to that of a series of curatively resected linitis plastica-type cancers, which often recur as peritoneal disease [47]. Furthermore, CY1 patients are deemed eligible for JCOG0501, a phase III trial to explore neoadjuvant chemotherapy by S-1 + cisplatin for scirrhous type gastric cancer in which the standard treatment arm consists of standard gastrectomy followed by S-1.

These facts indicate that CY1 patients could be indicated for the strategy consisting of standard gastrectomy and perioperative chemotherapy. In addition, S-1 monotherapy could be recommended for patients whose CY1 status was informed after gastrectomy. On the other hand, if the information on CY status was available prior to surgery, a chemotherapy-first strategy could be taken whereby only patients whose cytology status turned negative could be indicated for surgery [48, 49]. However, details of the optimal multidisciplinary treatment strategy in this setting, including the chemotherapeutic regimen to be used and the number of cycles to be delivered, remain to be elucidated in future clinical trials.

*CQ4*. Which chemotherapeutic regimen is recommended when recurrence was detected during or within 6 months from completion of the postoperative adjuvant chemotherapy with S-1?

*Answer* Although no evidence to recommend any particular regimen exists, most physicians would avoid monotherapy with S-1 for second-line chemotherapy.

Postoperative adjuvant chemotherapy with S-1 has been established as a standard of care for p-Stage II/III gastric cancer by the ACTS-GC trial. However, the treatment for patients who had recurrent disease after the adjuvant treatment remains to be elucidated.

The response rate of treatment by S-1 + cisplatin is reportedly low (5 %) for patients who had recurrence within 6 months from completion of the S-1 adjuvant therapy when compared with the response rate for recurrences after 6 months from the completion (37.5 %) [50]. This result, found in a multi-institutional retrospective analysis, suggests that cancers that recur during or early after completion of an adjuvant chemotherapy are resistant to the drug used in that chemotherapy. On the other hand, a retrospective analysis of the patients registered for the ACTS-GC study revealed that patients who received S-1 among other drugs in salvage line treatments survived longer after recurrence than those who did not receive S-1, regardless of the time interval between the adjuvant chemotherapy and recurrence. However, results of this study will have to be interpreted with caution since the study suffers from several biases in the background of the patients such as whether oral food intake was possible (patients who did not receive S-1 after recurrence might have been those with bowel obstruction who were unable to eat and suffered from poor performance status).

In treatment for colorectal cancer during the era of adjuvant chemotherapy with 5FU alone, drugs used in the salvage line treatment depended on the time interval between the completion of the adjuvant chemotherapy and recurrence. New regimens have been developed as first-line therapy for patients who had recurrence more than 6 months after completion of the adjuvant treatment and as second line for those who had recurrence during or within 6 months of the adjuvant therapy.

The same rule has been applied for gastric cancer, and patients with late recurrence after adjuvant treatment have been deemed eligible for clinical trials exploring a first-line treatment, whereas those with early recurrences were registered in clinical trials for the second-line treatment.

Thus, patients with recurrences during or early after completion of the adjuvant treatment are considered as targets of second-line treatments, and S-1 monotherapy is usually avoided for this population. However, there is currently no evidence to recommend any specific regimen for this setting.

*CQ5*. Which chemotherapeutic regimen is recommended for patients suffering from either bowel obstruction or massive ascites due to severe peritoneal metastases?

*Answer* The indication for chemotherapy itself should be decided discreetly, taking into consideration the general status of the patient. Drugs with mild toxicity profiles such as infusional 5-fluorouracil and paclitaxel could be considered as the candidates.

Standard of care has not been established for this population since the patients have not been eligible for most clinical trials for advanced/metastatic gastric cancer. Most patients in this population suffer from poor general status and will not tolerate the S-1 + cisplatin combination. Benefit for delivering chemotherapy should be weighed carefully against the risk, and best supportive care should be considered as an alternative.

The JCOG0106 study was one of the few in which only patients with peritoneal metastases detected by imaging studies such as CT and barium enema were eligible. In this trial, sequential therapy combining methotrexate + 5FU, which had been considered promising in this setting, was explored with continuous intravenous administration of 5FU (5FUci) as a control, but failed to show a survival benefit, while infusional 5FU was found to be less toxic [51]. Moreover, 5FUci enabled oral food intake in 41 % (7/27) of patients who had been unable to eat at the time of entry to the trial. Thus, 5FUci will be the current first choice for patients with bowel obstruction due to peritoneal metastases, but its effect on massive ascites remains elusive. On the other hand, another domestic phase III trial (ISO-5FU10) has shown non-inferiority of the 5FU + leucovorin (LV) combination against S-1 [52]. Thus, 5FU + LV, which can be delivered in the outpatient clinic, is another option for patients with relatively good general status.

In a phase II trial exploring weekly administration of paclitaxel in gastric cancer patients with ascites, improvement in the volume of ascites evaluated by a five-point measurement using the CT image was seen in 39 % (25/64) of the patients [53]. In a randomized phase II trial comparing second-line treatment by weekly administration of paclitaxel with the best available 5FU (either 5FUci or MTX + 5FU, which was not used in the first-line treatment) in patients with peritoneal metastases, a benefit in progression-free survival was proven, but no difference was detected in overall survival. However, paclitaxel was associated with a more favorable toxicity profile [54]. These results indicate that weekly paclitaxel can be considered for patients with severe peritoneal disease in both the first- and second-line setting. In addition, a phase II trial of the FLTAX regimen, which is a combination of paclitaxel with 5FU + LV, has shown that this combination reduced ascites in 44 % of patients [55]. Further evidence through a randomized comparison of the combination with a single-agent treatment is awaited.

*CQ6* Which chemotherapeutic regimen is recommended for elderly patients with unresectable/advanced gastric cancer?

*Answer* S-1 + cisplatin could be recommended for fit patients, but utmost care should be taken since the elderly patients are generally vulnerable to the adverse events. S-1 monotherapy could be selected for more frail patients.

Although S-1 + cisplatin is the standard first-line treatment for unresectable/recurrent gastric cancer in Japan, only patients up to 74 years of age were eligible for the SPIRITS trial that generated this evidence, and only 17 % (50/298) of patients registered for this trial were actually 70 years of age or older. In a subset analysis stratified by age, the hazard ratio of treatment by S-1 + cisplatin versus S-1 monotherapy was 0.75 (95 % CI 0.61–0.92) for patients under 60 years of age (*n* = 111) as opposed to 0.98 (95 % CI 0.82–1.17) for those between 60 and 69 and 0.95 (95 % CI 0.71–1.27) for those between 70 and 74 [15]. Thus, a benefit of adding cisplatin is unclear for the elderly population. In fact, there was no difference in survival between patients treated by S-1 + cisplatin and those treated by S-1 monotherapy in a retrospective study of the elderly population of ≥70 years of age, despite the apparent bias that the seemingly more fit patients were selected to receive the combination therapy [56]. Furthermore, a rather favorable outcome through S-1 monotherapy was reported in a cohort of patients ≥75 years of age in a prospective phase II trial focusing on elderly patients [57].

In short, regarding chemotherapy for advanced/metastatic gastric cancer, the evidence generated by the general population is unlikely to be directly applicable to elderly patients. That said, it may still be inadequate to estimate the tolerability of elderly patients to chemotherapy based only on chronological age without taking into account the major organ functions, comorbidities and past history. Unfortunately, however, a method to comprehensively evaluate the vulnerability of each aged individual has not been established.

Further evidence through clinical trials is needed for various decision-makings when treating elderly patients with gastric cancer. Until then, whether or not to deliver S-1 + cisplatin to these patients will have to be decided on a patient-by-patient basis based on the experience of each physician. Such decision will have to be based on the general condition of the patient with particular attention to the renal and cardiac function, always bearing in mind that S-1 monotherapy is quite reasonable as an alternative. Even after the treatment has started, the patient will have to be monitored with upmost care with attention paid not only to severe adverse events but also to anorexia, stomatitis and diarrhea, which could be particularly debilitating for elderly patients.

*CQ7*. Which chemotherapeutic regimen is recommended as a second-line treatment for HER2-positive gastric cancer?

*Answer* The taxanes or irinotecan can be recommended as in the case of HER2-negative cancer. However, in case a trastuzumab-containing regimen was not given as a first-line treatment, a combination of weekly paclitaxel and trastuzumab could be selected.

A trastuzumab-containing regimen is recommended for the first-line treatment of HER2-positive gastric cancer as a result of the ToGA trial [17]. There is no evidence to recommend any specific regimen for the specific cohort of HER2-positive patients who progressed during or after the trastuzumab-containing regimen. Either the taxanes or irinotecan could be selected as in the case of second-line treatment for HER2-negative gastric cancer.

On the other hand, a promising response rate of 37.0 % (95 % CI 23.2–52.5) and disease control rate of 82.6 % (95 % CI 68.2–92.2) were reported in a phase II trial exploring paclitaxel (weekly administration) + trastuzumab (JFMC45-1102 trial) for 46 evaluable patients with HER2-positive gastric cancer who were pretreated with a regimen that did not contain trastuzumab [58]. However, results of this trial will have to be interpreted with care for the following reasons: (1) Patients registered for this trial were not a typical cohort that receives second-line chemotherapy after the first-line treatment with a combination of fluorouracil and platinum agent in that 12 patients (26 %) were pretreated only with postoperative adjuvant chemotherapy while 5 patients (11 %) had already been treated with two lines of treatment; (2) although post-treatment cardiac function tests revealed that only one patient showed >10 % reduction in the left ventricular ejection fraction, other safety data are currently under analysis and have not been published.

There is currently no evidence in support of efficacy or safety for continuing with trastuzumab in case the patient was pretreated with a trastuzumab-containing regimen (trastuzumab beyond progression).

## References

[1] Japanese Gastric Cancer Association (2011). Japanese classification of gastric carcinoma: 3rd English edition. Gastric Cancer.

[2] Kaminishi M, Yamaguchi H, Shimizu N (1997). Stomach-partitioning gastrojejunostomy for unresectable gastric carcinoma. Arch Surg.

[3] Fujitani K, Yang HK, Mizusawa J (2016). Gastrectomy plus chemotherapy versus chemotherapy alone for advanced gastric cancer with a single non-curable factor (REGATTA): a phase 3, randomised controlled trial. Lancet Oncol.

[4] Sano T, Sasako M, Mizusawa J, et al. Randomized controlled trial to evaluate splenectomy in total gastrectomy for proximal gastric carcinoma. Ann Surg. 2016 (**Epub ahead of print**). 10.1097/SLA.0000000000001814 27280511

[5] Sasako M, Sano T, Yamamoto S (2008). D2 lymphadenectomy alone or with para-aortic nodal dissection for gastric cancer. N Engl J Med.

[6] Tokunaga M, Ohyama S, Hiki N (2009). Therapeutic value of lymph node dissection in advanced gastric cancer with macroscopic duodenum invasion: is the posterior pancreatic head lymph node dissection beneficial?. Ann Surg Oncol.

[7] Fujita J, Kurokawa Y, Sugimoto T (2012). Survival benefit of bursectomy in patients with resectable gastric cancer: interim analysis results of a randomized controlled trial. Gastric Cancer.

[8] Sasako M, Sano T, Yamamoto S (2006). Left thoracoabdominal approach versus abdominal-transhiatal approach for gastric cancer of the cardia or subcardia: a randomised controlled trial. Lancet Oncol.

[9] Katai H, Sasako M, Fukuda H (2010). Safety and feasibility of laparoscopy-assisted distal gastrectomy with suprapancreatic nodal dissection for clinical stage I gastric cancer: a multicenter phase II trial (JCOG 0703). Gastric Cancer.

[10] Nakamura K, Katai H, Mizusawa J (2013). A phase III study of laparoscopy-assisted versus open distal gastrectomy with nodal dissection for clinical stage IA/IB gastric Cancer (JCOG0912). Jpn J Clin Oncol.

[11] Kim HH, Hyung WJ, Cho GS (2010). Morbidity and mortality of laparoscopic gastrectomy versus open gastrectomy for gastric cancer: an interim report-a phase III multicenter, prospective, randomized Trial (KLASS Trial). Ann Surg.

[12] Gotoda T, Yanagisawa A, Sasako M (2000). Incidence of lymph node metastasis from early gastric cancer: estimation with a large number of cases at two large centers. Gastric Cancer.

[13] Hirasawa T, Gotoda T, Miyata S (2009). Incidence of lymph node metastasis and the feasibility of endoscopic resection for undifferentiated-type early gastric cancer. Gastric Cancer.

[14] Takizawa K, Kawata N, Tanaka M (2013). Treatment for intramucosal gastric cancer with mixed type histology (differentiated and undifferentiated). Stomach Intest.

[15] Koizumi W, Narahara H, Hara T (2008). Randomized phase III study of S-1 alone versus S-1=+ cisplatin in the treatment for advanced gastric cancer (The SPIRITS trial) SPIRITS: S-1 plus cisplatin vs S-1 in RCT in the treatment for stomach cancer. Lancet Oncol.

[16] Boku N, Yamamoto S, Shirao K (2009). Fluorouracil versus combination of irinotecan plus cisplatin versus S-1 in metastatic gastric cancer: a randomized phase 3 study. Lancet Oncol.

[17] Bang YJ, Van Cutsem E, Feyereislova A (2010). Trastuzumab in combination with chemotherapy versus chemotherapy alone for treatment of HER2-positive advanced gastric or gastro-oesophageal junction cancer (ToGA): a phase 3, open-label, randomised controlled trial. Lancet.

[18] Ohtsu A, Shah MA, Van Cutsem E (2011). Bevacizumab in combination with chemotherapy as first-line therapy in advanced gastric cancer: a randomized, double-blind, placebo-controlled phase III study. J Clin Oncol.

[19] Koizumi W, Kim YH, Fujii M (2014). Addition of docetaxel to S-1 without platinum prolongs survival of patients with advanced gastric cancer: a randomized study (START). J Cancer Res Clin Oncol.

[20] Narahara H, Ishii H, Imamura H (2011). Randomized phase III study comparing the efficacy and safety of irinotecan plus S-1 with S-1 alone as first-line treatment for advanced gastric cancer (study GC0301/TOP-002). Gastric Cancer.

[21] Cutsem EV, Moiseyenko VM, Tjulandin S (2006). Phase III study of docetaxel with cisplatin and 5-fluorouracil as first-line therapy for advanced gastric cancer: a report of the V325 study group. J Clin Oncol.

[22] Yamada Y, Higuchi K, Nishikawa K (2015). Phase III study comparing oxaliplatin plus S-1 with cisplatin plus S-1 in chemotherapy-naïve patients with advanced gastric cancer. Ann Oncol.

[23] Cunningham D, Starling N, Rao S (2008). Capecitabine and oxaliplatin for advanced esophagogastric cancer. N Engl J Med.

[24] Thuss-Patience PC, Kretzschmar A, Bichev D (2011). Survival advantage for irinotecan versus best supportive care as second-line chemotherapy in gastric cancer: a randomized phase III study of the Arbeitsgemeinschaft Internistische Onkologie (AIO). Eur J Cancer.

[25] Kang JH, Lee SI, Lim DH (2012). Salvage chemotherapy for pretreated gastric cancer: a randomized phase III trial comparing chemotherapy plus best supportive care with best supportive care alone. J Clin Oncol.

[26] Hironaka S, Ueda S, Yasui H (2013). Randomized, open-label, phase III study comparing irinotecan with paclitaxel in patients with advanced gastric cancer without severe peritoneal metastasis after failure of prior combination chemotherapy using fluoropyrimidine plus platinum: WJOG 4007 trial. J Clin Oncol.

[27] Wilke H, Muro K, Van Cutsem E (2014). Ramucirumab plus paclitaxel versus placebo plus paclitaxel in patients with previously treated advanced gastric or gastro-oesophageal junction adenocarcinoma (RAINBOW): a double-blind, randomised phase 3 trial. Lancet Oncol.

[28] Fuchs CS, Tomasek J, Yong CJ (2014). Ramucirumab monotherapy for previously treated advanced gastric or gastro-oesophageal junction adenocarcinoma (REGARD): an international, randomised, multicenter, placebo-controlled, phase 3 trial. Lancet.

[29] Sakuramoto S, Sasako M, Yamaguchi T (2007). Adjuvant chemotherapy for gastric cancer with S-1, an oral fluoropyrimidine. N Engl J Med.

[30] Sasako M, Sakuramoto S, Katai H (2011). Five-year outcomes of a randomized phase III trial comparing adjuvant chemotherapy with S-1 versus surgery alone in stage II or III gastric cancer. J Clin Oncol.

[31] Takahari D, Hamaguchi T, Yoshimura K (2011). Feasibility study of adjuvant chemotherapy with S-1 plus cisplatin for gastric cancer. Cancer Chemother Pharmacol.

[32] Kodera Y, Ishiyama A, Yoshikawa T (2010). A feasibility study of postoperative chemotherapy with S-1 and cisplatin (CDDP) for gastric carcinoma (CCOG0703). Gastric Cancer.

[33] Noh SH, Park SR, Yang HK (2014). Adjuvant capecitabine plus oxaliplatin for gastric cancer after D2 gastrectomy (CLASSIC): 5-year follow-up of an open-label, randomised phase 3 trial. Lancet Oncol.

[34] Fuse N, Bando H, Chin K (2016). Adjuvant capecitabine plus oxaliplatin after D2 gastrectomy in Japanese patients with gastric cancer: a phase II study. Gastric Cancer.

[35] Shitara K, Chin K, Yoshikawa T (2015). Phase II study of adjuvant chemotherapy of S-1 plus oxaliplatin for patients with stage III gastric cancer after D2 gastrectomy. Gastric Cancer.

[36] Roviello F, Pedrazzani C, Marrelli D (2010). Super-extended (D3) lymphadenectomy in advanced gastric cancer. Eur J Surg Oncol.

[37] Tsuburaya A, Mizusawa J, Tanaka Y (2014). Neoadjuvant chemotherapy with S-1 and cisplatin followed by D2 gastrectomy with para-aortic lymph node dissection for gastric cancer with extensive lymph node metastasis. Br J Surg.

[38] Yoshida M, Ohtsu A, Boku N (2004). Long-term survival and prognostic factors in patients with metastatic gastric cancers treated with chemotherapy in the Japan Clinical Oncology Group (JCOG) study. Jpn J Clin Oncol.

[39] Park IH, Kim SY, Kim YW (2011). Clinical characteristics and treatment outcomes of gastric cancer patients with isolated para-aortic lymph node involvement. Cancer Chemother Pharmacol.

[40] Sakamoto Y, Sano T, Shimada K (2007). Favorable indications for hepatectomy in patients with liver metastasis from gastric cancer. J Surg Oncol.

[41] Takemura N, Saiura A, Koga R (2012). Long-term outcomes after surgical resection for gastric cancer liver metastasis: an analysis of 64 macroscopically complete resections. Langenbecks Arch Surg.

[42] Cheon SH, Rha SY, Jeung HC (2008). Survival benefit of combined curative resection of the stomach (D2 dissection) and liver in gastric cancer patients with liver metastases. Ann Oncol.

[43] Kodera Y, Fujitani K, Fukushima N (2014). Surgical resection of hepatic metastasis from gastric cancer: a review and new recommendation in the Japanese gastric cancer treatment guidelines. Gastric Cancer.

[44] Bando E, Yonemura Y, Takeshita Y (1999). Intraoperative lavage for cytological examination in 1297 patients with gastric carcinoma. Am J Surg.

[45] Kodera Y, Ito S, Mochizuki Y (2012). Long-term follow up of patients who were positive for peritoneal lavage cytology: final report from the CCOG0301 study. Gastric Cancer.

[46] Bando E, Makuuchi R, Miki Y, et al. Clinical significance of intraoperative peritoneal cytology in gastric carcinoma-analysis of 3124 patients. 10th International Gastric Cancer Congress 2013: Abstract, P27-5.

[47] Kinoshita T, Sasako M, Sano T (2009). Phase II trial of S-1 for neoadjuvant chemotherapy against scirrhous gastric cancer (JCOG0002). Gastric Cancer.

[48] Okabe H, Ueda S, Obama K (2009). Induction chemotherapy with S-1 plus cisplatin followed by surgery for treatment of gastric cancer with peritoneal dissemination. Ann Surg Oncol.

[49] Mezhir JJ, Shah MA, Jacks LM (2010). Positive peritoneal cytology in patients with gastric cancer: natural history and outcome of 291 patients. Ann Surg Oncol.

[50] Shitara K, Morita S, Fujitani K (2012). Combination chemotherapy with S-1 plus cisplatin for gastric cancer that recurs after adjuvant chemotherapy with S-1: multi-institutional retrospective analysis. Gastric Cancer.

[51] Shirao K, Boku N, Yamada Y (2013). Randomized phase III study of 5-fluorouracil continuous infusion vs. sequential methotrexate and 5-fluorouracil therapy in far advanced gastric cancer with peritoneal metastasis (JCOG0106). Jpn J Clin Oncol.

[52] Sawaki A, Yamaguchi K, Nabeya Y (2009). 5FU/l-LV (RPMI) versus S-1 as first-line therapy in patients with advanced gastric cancer: a randomized phase III non-inferiority trial (ISO-5FU10 Study Group trial). Eur J Cancer.

[53] Imamoto H, Oba K, Sakamoto J (2011). Assessing clinical benefit response in the treatment of gastric malignant ascites with non-measurable lesions: a multicenter phase II trial of paclitaxel for malignant ascites secondary to advanced/recurrent gastric cancer. Gastric Cancer.

[54] Nishina H, Boku K, Gotoh J (2016). Randomized phase II study of second-line chemotherapy with the best available 5-fluorouracil regimen versus weekly administration of paclitaxel in far advanced gastric cancer with severe peritoneal metastases refractory to 5-fluorouracil-containing regimens (JCOG0407). Gastric Cancer.

[55] Iwasa S, Goto M, Yasui H (2012). Multicenter feasibility study of combination therapy with fluorouracil, leucovorin and paclitaxel (FLTAX) for peritoneal disseminated gastric cancer with massive ascites or inadequate oral intake. Jpn J Clin Oncol.

[56] Tsushima T, Hironaka S, Boku N (2013). Comparison of safety and efficacy of S-1 monotherapy and S-1 plus cisplatin therapy in elderly patients with advanced gastric cancer. Int J Clin Oncol.

[57] Koizumi W, Akiya T, Sato A (2010). Phase II study of S-1 as first-line treatment for elderly patients over 75 years of age with advanced gastric cancer: the Tokyo Cooperative Oncology Group study. Cancer Chemother Pharmacol.

[58] Isawa S, Nishikawa K, Miki A (2013). Multicenter, phase II study of trastuzumab and paclitaxel to treat HER2-positive, metastatic gastric cancer patients naïve to trastuzumab (JFMC45-1102). J Clin Oncol.

